# A matched-pair case control study identifying hemodynamic predictors of cerebral aneurysm growth using computational fluid dynamics

**DOI:** 10.3389/fphys.2023.1300754

**Published:** 2023-12-15

**Authors:** Allyson J. Weiss, Aaron O. Panduro, Erica L. Schwarz, Zachary A. Sexton, Ingrid S. Lan, Thomas. R. Geisbush, Alison L. Marsden, Nicholas A. Telischak

**Affiliations:** ^1^ Department of Mechanical Engineering, Stanford University, Stanford, CA, United States; ^2^ Department of Biochemistry, California State University, Fresno, CA, United States; ^3^ Department of Bioengineering, Stanford University, Stanford, CA, United States; ^4^ Department of Radiology, School of Medicine, Stanford University, Stanford, CA, United States; ^5^ Department of Pediatrics, School of Medicine, Stanford University, Stanford, CA, United States

**Keywords:** aneurysm, cerebral, blood flow, stroke, intracranial, computational fluid dynamics, hemodynamics

## Abstract

**Introduction:** Initiation and progression of cerebral aneurysms is known to be driven by complex interactions between biological and hemodynamic factors, but the hemodynamic mechanism which drives aneurysm growth is unclear. We employed robust modeling and computational methods, including temporal and spatial convergence studies, to study hemodynamic characteristics of cerebral aneurysms and identify differences in these characteristics between growing and stable aneurysms.

**Methods:** Eleven pairs of growing and non-growing cerebral aneurysms, matched in both size and location, were modeled from MRA and CTA images, then simulated using computational fluid dynamics (CFD). Key hemodynamic characteristics, including wall shear stress (WSS), oscillatory shear index (OSI), and portion of the aneurysm under low shear, were evaluated. Statistical analysis was then performed using paired Wilcoxon rank sum tests.

**Results:** The portion of the aneurysm dome under 70% of the parent artery mean wall shear stress was higher in growing aneurysms than in stable aneurysms and had the highest significance among the tested metrics (*p* = 0.08). Other metrics of area under low shear had similar levels of significance.

**Discussion:** These results align with previously observed hemodynamic trends in cerebral aneurysms, indicating a promising direction for future study of low shear area and aneurysm growth. We also found that mesh resolution significantly affected simulated WSS in cerebral aneurysms. This establishes that robust computational modeling methods are necessary for high fidelity results. Together, this work demonstrates that complex hemodynamics are at play within cerebral aneurysms, and robust modeling and simulation methods are needed to further study this topic.

## 1 Introduction

Approximately 5% of the population develops at least one cerebral aneurysm in their lifetime. Of these, about 0.2% rupture every year, causing subarachnoid hemorrhages which carry a mortality rate of almost 50% (Sadasivan et al., 2013). Because of the significant risk of mortality, identifying indicators for both aneurysm growth and rupture is of critical importance in the clinical setting.

Historically, clinicians have commonly used morphological characteristics such as size and shape to assess the risk of rupture. However, hemodynamics also play a significant role in aneurysm development. Multiple studies have shown that initiation and progression of cerebral aneurysms is driven by complex interactions between biological and hemodynamic factors, one hemodynamic factor being wall shear stress (WSS), and that rupture occurs when the stress on the wall exceeds the wall’s strength (Meng et al., 2007; Metaxa et al., 2010). However, the hemodynamic mechanism that drives aneurysm growth is still under debate. Elevated maximal WSS can cause endothelial injury, initiating wall remodeling and potential degeneration and therefore aneurysm initiation and growth (Sforza et al., 2009; Meng et al., 2014). Conversely, regions of low velocity within aneurysms can result in low WSS, which can lead to wall inflammation along with localized degeneration and thinning (Sforza et al., 2009; Meng et al., 2014).

Computational fluid dynamics (CFD) offers a unique opportunity to study patient-specific hemodynamics in cerebral arteries and aneurysms by simulating *in-vivo* blood flow through imaging-derived geometrical models. While previous studies have investigated cerebral aneurysms using CFD, most assumed traction-free outlet boundary conditions (Shojima MD et al., 2004; Mut et al., 2011; Xiang et al., 2011; Kono et al., 2012; Fukazawa et al., 2015). Under traction-free conditions, the outlet face is free from external stress; however, a physiological cerebral artery is not pressure-free, and can even experience time-varying changes in outlet resistance, making these models physiologically inaccurate.

In addition to the assumption of constant resistance, multiple studies also split outflow volumes according to Murray’s law (Sforza et al., 2016; Brinjikji et al., 2017; Chung et al., 2018). However, previously reported values of cerebral flow splits deviate substantially from Murray’s law, suggesting that cerebral arteries are potentially not well governed by Murray’s law (Seymour et al., 2020). Further, previous studies that split flows by Murray’s law fail to consider temporal variations in downstream resistance, which also leads to physiologically inaccurate flows. Thus, the lack of physiological boundary conditions warrants a study with higher physiological detail to more closely model the patients hemodynamics. The variable waveform shape can be more accurately governed by RCR boundary conditions to account for downstream capacitance (Vignon-Clementel et al., 2010).

To investigate the association between hemodynamics and cerebral aneurysm growth and address previous studies’ limitations, we analyze 11 pairs of growing and non-growing aneurysms pair-matched both in size and location. We use pulsatile inflow waveforms and the Windkessel (RCR) outlet boundary conditions to create physiological simulations. In addition, we perform mesh refinement to ensure WSS convergence and investigate the effect of mesh sizing on hemodynamic fields of interest. We then extract hemodynamic metrics in the aneurysm dome as well as in the parent artery and perform statistical analysis on the matched pairs.

## 2 Materials and methods

### 2.1 Data collection

**FIGURE 1:**
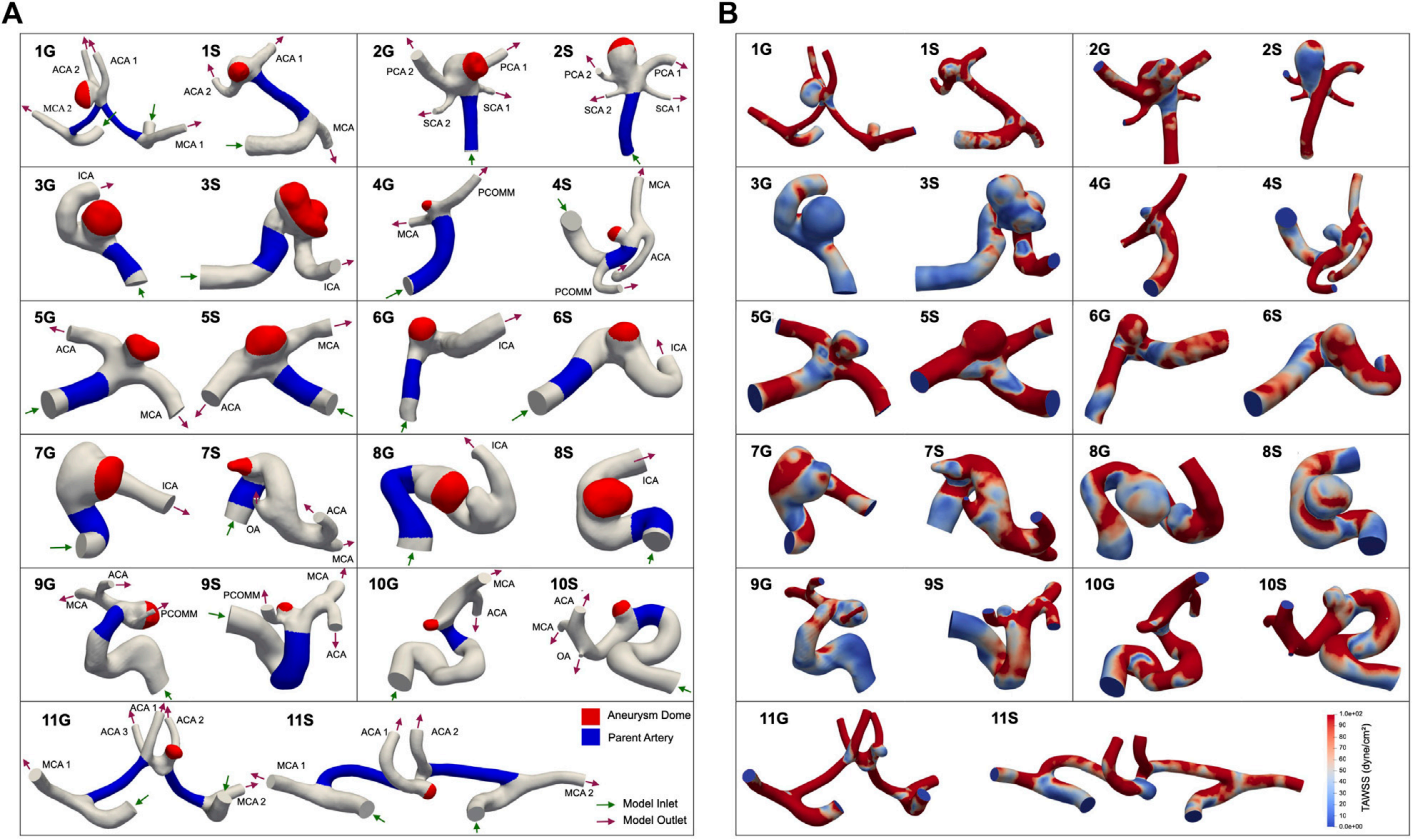
11 pairs of aneurysms in our cohort, matched in both size and location. ‘G’ denotes a growing aneurysm, and ‘S’ denotes a stable aneurysm. The aneurysm dome is represented by the red region, and the parent artery is represented by the blue region **(A)**. The aneurysm dome was clipped to exclude the impingement region, which is defined as the region where flow impinges against the aneurysm wall (Cebral et al., 2005b). **(B)** Shows time-averaged WSS distributions, in dyne/cm^2^. Abbreviations: ACA, anterior cerebral crtery; ICA, internal carotid artery; MCA, middle cerebral artery; OA, ophthalmic artery; PCA, posterior cerebral artery; PCOMM, posterior communicating artery; SCA, superior cerebellar artery.

CT angiography (CTA) and MR angiography (MRA) reports from 2002 to 2021 were retrospectively queried through a Stanford Health database as part of a study approved by the Stanford University Institutional Review Board (IRB). From these reports, patient aneurysms were identified that had undergone a follow-up scan more than 1 year after initial imaging. Subsequently, the selected aneurysms were measured in three orthogonal dimensions, and volumetric estimates were calculated based on these measurements. Aneurysms were then categorized as either growing or stable and matched both by location and volume. Growing aneurysms were characterized by an increase in size of at least 1 mm in two or more dimensions on a subsequent scan, while measurements for stable aneurysms remained within a 1 mm increase in all dimensions. A total of 11 growing aneurysms were identified and paired with stable aneurysms possessing similar volume and the same location for inclusion in this study (refer to Figure 1A for models). Demographic information for the selected patients is available in Table 1 below.

**TABLE 1:** Patient demographics and aneurysm characteristics.

	Stable aneurysm	Growing aneurysm
Patients (number)	11	11
Age, Y, Mean ± SD	65 ± 9	61 ± 10
Female, N (%)	9 (82%)	9 (82%)
Medical Comorbidities		
Hypertension	5 (45%)	3 (27%)
Hyperlipidemia	5 (45%)	3 (27%)
Diabetes Mellitus	2 (18%)	1 (9%)
Social and Family History		
Smoker History	3 (27%)	7 (63%)
Family History of Aneurysm	2 (18%)	1 (9%)
Aneurysm Location		
Cavernous ICA	4	4
Supraclinoid/ICA Terminus	2	2
Anterior Communicating	2	2
Posterior Communicating	2	2
Basilar Apex	1	1
Size		
Median Size (mm^3^) ± IQR	443.5 ± 557.7	371.4 ± 761.2
Median Time Between Scans (years) ± IQR	4.6 ± 5.5	3.4 ± 3.4

### 2.2 Patient-specific model construction

Patient-specific, three-dimensional anatomical models of each cerebral aneurysm (Figure 1) were constructed from MRA and CTA images using the level set segmentation tool in the Vascular Modeling Toolkit, which is a collection of tools for image-based 3D reconstruction, geometric analysis, and surface data processing of blood vessels. Methodology details are provided at www.vmtk.org. Each segment was modeled by placing two seeds on the segment of interest and subsequently propagating wavefronts based on the contrasting image intensity of the vessel and surrounding tissue to create a tubular vessel model. Once segmented, each model was smoothed using Autodesk Meshmixer (www.meshmixer.com). Inlets and outlets were truncated perpendicular to the vessel using ParaView (Ahrens et al., 2005). All models were then imported into the open-source software SimVascular, a software package that provides a pipeline from medical image segmentation to blood flow simulation and analysis. Methodology details are provided at simvascular.github.io. They were then meshed with linear tetrahedral elements using TetGen (Research Group Numerical Mathematics and Scientific Computing, 2015) and an initial maximum edge size informed by the minimum outlet size (approximately 0.25–0.4 mm). All models used in this study can be located in the Vascular Model Repository, an open-source database of vascular models, at vascularmodel.com.

### 2.3 Simulation

All hemodynamic simulations were performed using svSolver, SimVascular’s finite element simulation suite. Wall compliance was neglected, and no-slip boundary conditions were applied at the walls. Blood was approximated as a Newtonian fluid with density 1.06 g/cm^3^ and viscosity 0.04 Poise, with the assumption of a constant viscosity based on previous studies that indicate that the Newtonian fluid assumption is generally acceptable in CFD of cerebral aneurysms (Fisher and Stroud Rossmann, 2009; Xiang et al., 2012).

**FIGURE 2:**
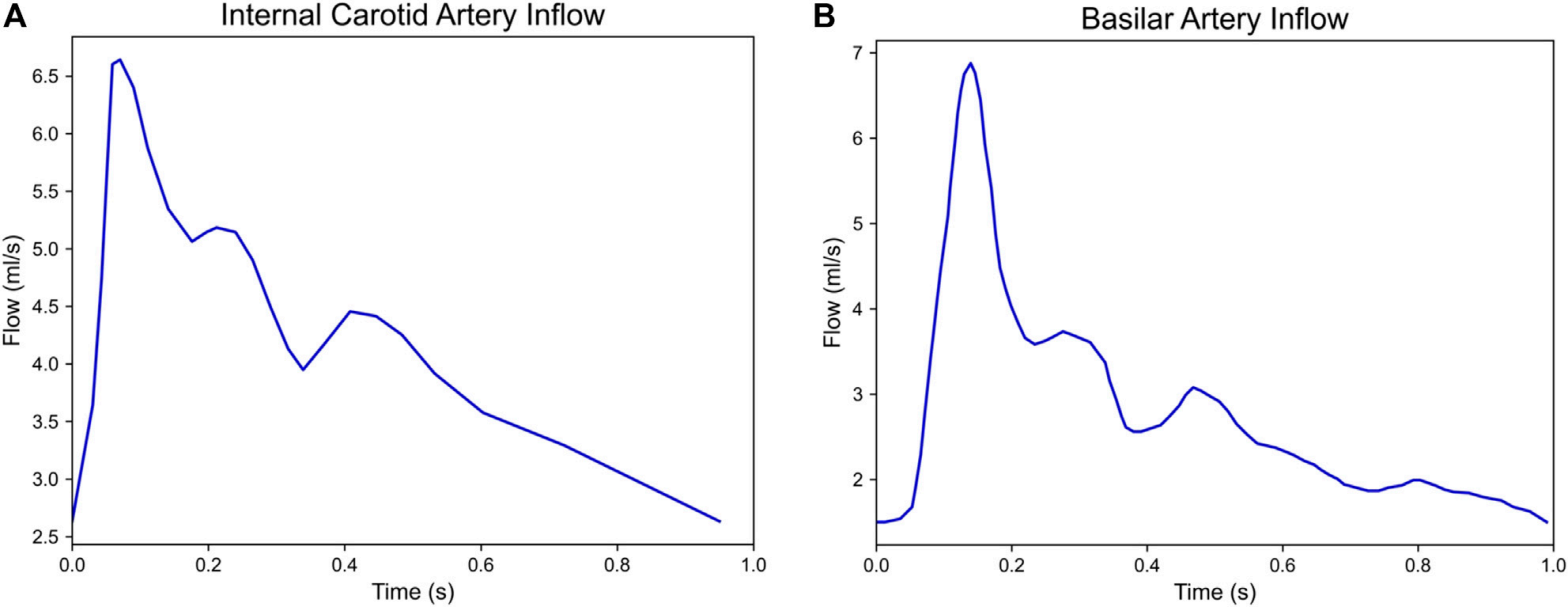
Flow waveforms of the **(A)** internal carotid artery (ICA) and **(B)** basilar artery (BA) prescribed at the inlets of our simulated models. 10 pairs of growing and stable aneurysms required the ICA inflow and 1 pair (2G and 2S) required the BA inflow.

Clinically measured flow waveforms were prescribed at the inlets with a parabolic velocity profile. The waveforms reference flow rates and waveforms of healthy patients reported in previous studies (Gwilliam et al., 2009; Wake-Buck et al., 2012; Seymour et al., 2020) and can be seen in Figure 2. RCR Windkessel boundary conditions were applied to each outlet (Supplementary Table S1), with the outlet resistances split inversely to the outlet areas, as in the following equation:

$$R_{i}=R_{total}*\frac{A_{total}}{A_{i}},$$

where $A_{i}$ is the area of outlet *i,* $A_{total}$ is the summation of all outlet areas, $A_{total}=\sum_{i=1}^{n}A_{i}$ , and *n* is the total number of outlets (Figure 1).

The ratio of proximal to distal resistance in the RCR circuit was prescribed as 1:9. The resistance and capacitance values were tuned to achieve target systolic and diastolic aortic pulse pressures within 5% of 120 mmHg and 80 mmHg, respectively. 3D simulations were run until inlet and outlet pressures were converged within 5% to a limit cardiac cycle. Upon completion of outlet boundary condition tuning, we performed mesh refinement to ensure time-averaged WSS (TAWSS) convergence.

### 2.4 Mesh convergence

Finite element solutions are sensitive to discretization schemes, with known dependence on mesh size. This is particularly true when seeking to analyze hemodynamic quantities, such as WSS, which are dependent on the spatial gradient of the velocity solution (Craven et al., 2018). To ensure spatial convergence of our hemodynamic quantities of interest, we performed a mesh convergence study on our aneurysm models. We began by discretizing our meshes with tetrahedral elements with a maximum edge size of 0.25–0.4 mm, dependent on the size of the aneurysm model. The spatially averaged TAWSS was then calculated in the aneurysm body. The maximum edge size of the model was then decreased by approximately 10% with every refinement iteration. The model was determined to be converged when the spatially averaged TAWSS in the dome was within 5% of that in the previous refinement.

### 2.5 Postprocessing

Following simulation of each aneurysm model, we postprocessed the results of the final cardiac cycle of the simulation. We segmented the model into two regions corresponding to the aneurysm dome and the parent vessel. Mean, minimum, and maximum TAWSS and oscillatory shear index (OSI) parameters were extracted from both the dome and parent regions (Figure 1A). TAWSS and OSI were calculated using the following equations:

$$TAWSS=\frac{\int_{0}^{T}\left|WS\right.S_{i}\left|dt\right.}{T}$$

and

$$OSI=\frac{1}{2}\left\{1-\frac{\left|\int_{0}^{T}WSS_{i}dt\right|}{\int_{0}^{T}\left|WSS_{i}\right|dt}\right\}.$$

The mean TAWSS in the parent artery was used to determine thresholds for identifying areas of low TAWSS in the aneurysm dome. We calculated the low shear area (LSA), defined as the non-dimensional ratio of the dome area with TAWSS at least one standard deviation below the mean TAWSS in the parent artery to the total dome area. We also calculated the non-dimensional area beneath a direct percentage of the parent artery mean TAWSS as the mean-thresholded low shear area (MTLSA_X%_) where the threshold value is defined as X% of mean parent artery TAWSS. The low shear area was calculated as a percentage of the parent artery mean TAWSS to accommodate varying flow patterns in patient-specific vessel geometries, as opposed to using an absolute threshold for all models which may not be applicable to each patient-specific geometry.

### 2.6 Statistical analysis

The mean, minimum, and maximum values of the TAWSS and OSI and low WSS exposure areas were calculated in the aneurysm dome for both the growing and non-growing aneurysms. A Shapiro-Wilk test determined that not all metrics demonstrated a normal distribution (Supplementary Table S2), so a paired Wilcoxon rank sum test was performed to determine the statistical significance of the computed differences between growing and non-growing aneurysms. We denote a significance of *p* = 0.1 in our reported results.

## 3 Results

### 3.1 Temporal convergence

Each simulation in our study was run to a limit cardiac cycle to ensure temporal convergence of pressure. Boundary conditions were tuned to achieve target systolic and diastolic pressures within 5% of 120 mmHg and 80 mmHg, respectively.

### 3.2 Mesh convergence

**FIGURE 3:**
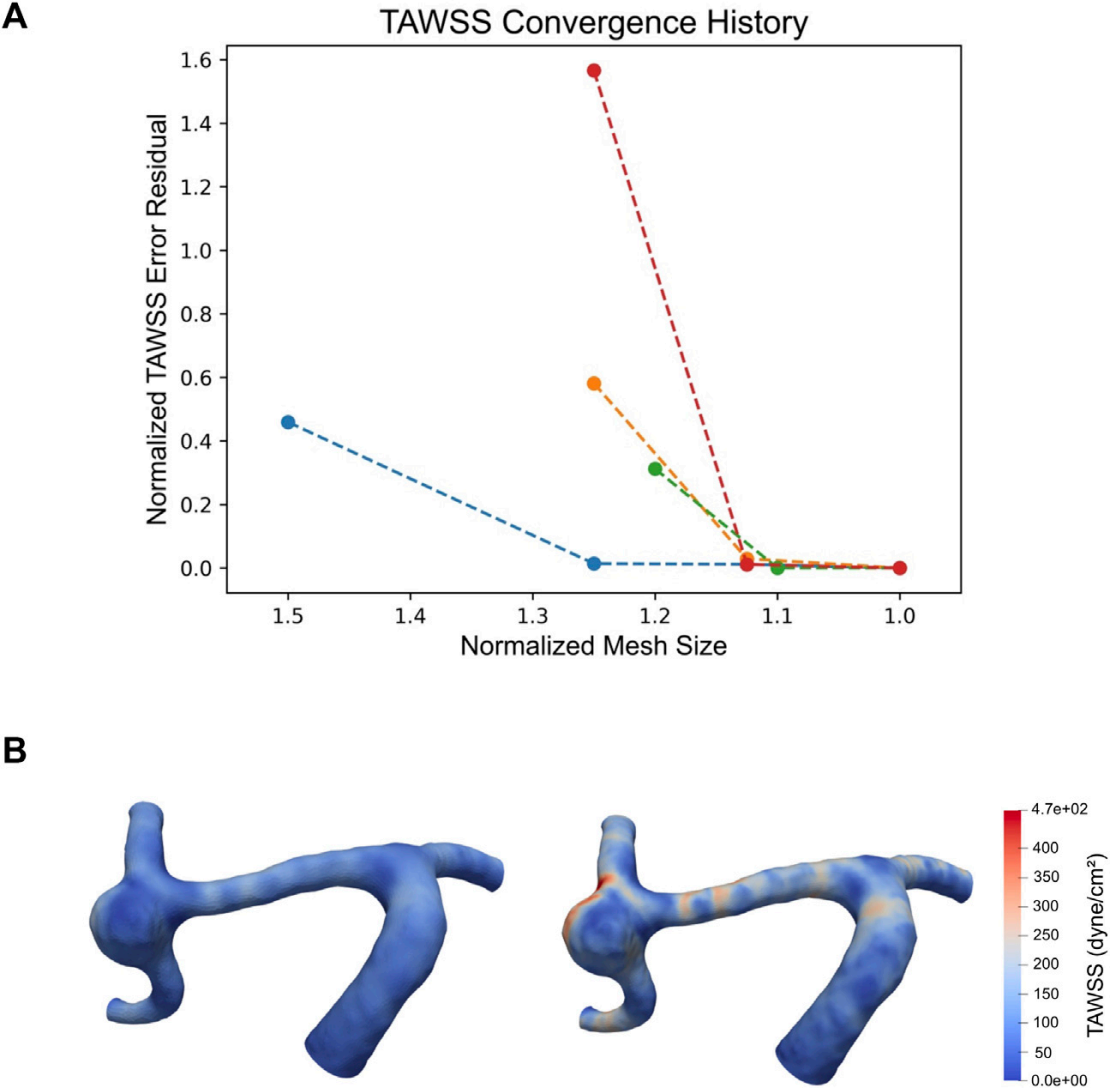
Plot of TAWSS convergence of cases 1S, 2G, 2S, and 3S, where each point represents a mesh refinement step. Normalized mesh size is defined as $\frac{mesh\;size\;}{final\;converged\;mesh\;size}$ , and normalized spatially averaged TAWSS error residual is defined as $\frac{\left|dome\;TAWSS-final\;dome\;TAWSS\right|}{final\;dome\;TAWSS}$ **(A)**. TAWSS distribution over aneurysm 1S (denoted by the blue line in the TAWSS convergence plot in Figure 2A): left, initial mesh refinement (aneurysm mean TAWSS = 8.518 dyne/cm^2^); right, final mesh refinement (aneurysm mean TAWSS = 15.754 dyne/cm^2^) **(B)**.

All cases converged within the specified 5% tolerance. The final maximum edge sizes ranged between 0.144 mm and 0.32 mm across models. Several models required multiple refinement iterations, resulting in large changes in TAWSS between refinements, including a maximum error of over 150% between the mean TAWSS of the initial model and the mean TAWSS of the final, refined model (Figure 3).

### 3.3 Stable versus growing aneurysms

We found differences with a statistical significance at *p* = 0.1 between growing and stable cerebral aneurysms for the portion of the aneurysm dome under 70% of the parent artery spatially-averaged TAWSS (Table 2). The portion of the aneurysm under 50% and 90% of the parent artery spatially averaged TAWSS showed marginal significances of *p* = 0.101 and 0.110, respectively (Table 2). Distributions of WSS, OSI, and pressures of each model can be found in Figure 1B and Supplementary Figures S1A, B, respectively.

**TABLE 2:** Mean and standard deviations of hemodynamic variables of interest for stable and growing aneurysms, and corresponding *p* values from the paired Wilcoxon rank sum test.

	Stable			Growing		*p* value
	Mean		SD	Mean	SD	
Mean TAWSS (dyne/cm^2^)	55.60		29.8	72.74	68.78	0.83
Max TAWSS (dyne/cm^2^)	140.08		61.2	186.50	134.19	0.41
Min TAWSS (dyne/cm^2^)	11.14		7.74	11.38	10.83	0.97
Mean OSI	0.03		0.05	0.01	0.01	0.41
Max OSI	0.27		0.12	0.25	0.08	0.83
Min OSI	0.002		0.003	0.0005	0.0004	0.41
LSA (%)	46.72		33.23	36.92	38.12	0.46
MTLSA_50%_ (%)	37.60		0.33	52.00	28.11	0.10
MTLSA_70%_ (%)	55.74		0.31	73.30	23.21	0.08^†^
MTLSA_90%_ (%)	69.81		0.26	87.22	17.14	0.11

TAWSS, time averaged wall shear stress; OSI, oscillatory shear index; LSA, low shear area; MTLSA, mean-thresholded low shear area.

## 4 Discussion

It is well established that WSS plays a role in cerebral aneurysm initiation and growth, and that when the stress exceeds the wall’s strength, rupture occurs (Metaxa et al., 2010; Meng et al., 2014). However, less is known about the hemodynamics of aneurysm growth. Recent studies have used CFD to assess several hemodynamic variables, including WSS and OSI, and their association with cerebral aneurysm growth and rupture. One study of seven aneurysms found that low TAWSS is associated with localized aneurysm wall deformation of at least 0.3 mm^3^, while another study of 33 cerebral aneurysms indicated that unstable aneurysms have higher concentrations of WSS and higher area of low WSS than growing aneurysms, though the latter was not found to be statistically significant (Sforza et al., 2016). Despite the identification of significant indicators, particularly WSS, within individual studies, the lack of consistently significant indicators of aneurysm growth highlights the complexity of the interactions between physiology, anatomy, genetics, and hemodynamics and demonstrates the need for additional research into the link between hemodynamics and aneurysm outcomes.

In our study of 11 pairs of growing and stable aneurysms matched in both size and location, we found differences with a statistical significance at *p* = 0.1 between aneurysm growth and the area of the aneurysm subjected to low WSS. Growing aneurysms tended to have a larger area of the aneurysm under 70% of the spatially averaged TAWSS in the parent artery. This finding indicates that CFD may be a useful prognostic tool in the clinical setting, beyond the current indicators of size and location, for assessing the risk of aneurysm progression. We also found that growing aneurysms had higher area under low shear stress across multiple threshold values (Table 2), which agrees with other papers in this field (Sforza et al., 2009; Brinjikji et al., 2017; Chung et al., 2018). Although we did not reach statistical significance at the *p* = 0.05 level, our results align with previously observed trends, which noted that growing aneurysms tend to have higher areas of low shear compared to their non-growing counterparts, although the exact definition of low shear and level of significance varied greatly between studies. Consistent with the low flow theory of aneurysm progression, a higher region of low shear in the aneurysm dome may lead to larger areas of localized degeneration and thinning, which can give rise to aneurysm growth due to thinner, weaker vessel walls (Sforza et al., 2009; Meng et al., 2014). Thus, when considered in context with the wider body of aneurysm growth research, particularly in the application of CFD, our findings indicate a promising direction for future study of the link between aneurysm growth and area under low shear.

CFD represents an important tool for investigating hemodynamic forces that are immeasurable *in-vivo*. However, utilizing CFD requires the careful selection of computational parameters for hemodynamic simulation, including boundary conditions, mesh size, and simulation duration (Vignon-Clementel et al., 2010; Craven et al., 2018). Pursuant to this, we utilized robust methods to ensure physiological and numerical accuracy in our CFD simulations. While many previous studies relied on Murray’s Law to establish flow splits, values for flow splits in cerebral arteries recorded in literature were not consistent with this assumption (Kobayashi and Karino, 2010; Seymour et al., 2020). Using area to distribute resistance has been shown to more consistently represent physiological conditions than Murray’s Law in cerebrovascular simulations (Chnafa et al., 2018), leading us to split resistance and flow based on outlet area. Many previous studies also utilized purely resistance-based outlet conditions, assuming zero capacitance in the distal vascular bed. This has been shown to be non-physiological and can affect the time-resolved velocity field, which in turn changes the calculated velocities and WSS (Vignon-Clementel et al., 2010). To address this, we utilized Windkessel RCR boundary conditions that incorporate distal capacitance and resistance and tuned the values to achieve physiological pressure and flow waveforms. Many previous studies also ran simulations of only 2 or 3 cardiac cycles (Cebral et al., 2005a; Boussel et al., 2008; Castro et al., 2009; Cebral et al., 2011; Mut et al., 2011; Xiang et al., 2011; Byrne et al., 2014; Sforza et al., 2016; Brinjikji et al., 2017; Chung et al., 2018), which we did not find to be enough to achieve pressure convergence in many cases. In this study, we ensured temporal convergence by running simulations until a limit cycle was reached.

Further, previous studies investigating CFD in growing vs. stable cerebral aneurysms reported using a constant mesh size across all models and did not perform mesh refinement to ensure spatial convergence of hemodynamic values such as WSS (Cebral et al., 2005a; Mut et al., 2011; Brinjikji et al., 2017). Previous papers have investigated the effect of mesh size on cerebral aneurysm flow simulations (Cebral et al., 2005b), however these did not look at the effect of mesh size on WSS specifically and only noted the effect of mesh size on overall flow features. As a gradient of the velocity field, it is known that WSS is more sensitive to mesh size in finite element models (Craven et al., 2018) and can require additional mesh refinement to achieve similar levels of convergence. Throughout our mesh adaptation studies, we saw that WSS was highly sensitive to changes in mesh size, and we even observed changes in WSS greater than 150% between refinement steps (Figure 3). This has significant implications on the accuracy of WSS in studies that forgo mesh convergence and indicates that mesh convergence is necessary to have strong confidence in the accuracy of reported WSS values. By conducting a WSS convergence study, we can be confident that the WSS values we calculate accurately represent the hemodynamic stresses inside of our modeled geometries.

There remain several limitations in our study. We modeled our blood as an incompressible Newtonian fluid. Some studies indicate that the assumption of blood as a Newtonian fluid may be a significant oversimplification of hemodynamics, but a clear consensus has not yet been reached about the importance of non-Newtonian effects on the simulation of cerebral aneurysms, or which non-Newtonian model should be selected. Some studies found that hemodynamic differences are less sensitive to blood constitutive law than they are to aneurysm geometry (Fisher and Stroud Rossmann, 2009), while others suggest that the Newtonian assumption could overestimate wall shear stress in low-flow regions (Xiang et al., 2012). However, both studies came to the conclusion that the Newtonian fluid assumption is generally acceptable in CFD of cerebral aneurysms (Fisher and Stroud Rossmann, 2009; Xiang et al., 2012).

We also assumed rigid vessel walls due to the lack of information regarding vessel wall thickness and mechanical properties. A CFD study on the influence of wall compliance on hemodynamics in intracranial aneurysms observed an overestimation of WSS magnitude in rigid models, but a number of other hemodynamic characteristics were not found to vary significantly between rigid models and compliant models (Dempere-Marco et al., 2006). Although there may be differences in CFD analysis of rigid and compliant vessels, the variation of wall motion among patients is unknown (Dempere-Marco et al., 2006), as are the wall compliance properties of our modeled aneurysms. For this reason, we assumed a rigid wall for all of our models.

Another limitation was utilizing the same flow profile across our patient cohort. While there may be differences in cerebral inflow among patients, patient-specific flows are rarely measured in practice due to the additional time required to obtain the measurements and the difficulty of perpendicular slice selection at desired locations as a result of the tortuous nature of cerebral vascular (Gwilliam et al., 2009). The lack of clinically measured, patient-specific flow profiles for each patient in our study necessitated this assumption. Alternative approaches may be considered in the future, including scaling the waveform based on allometric assumptions of cardiac output. Another approach to account for lack of patient-specific waveforms may be to couple the 3D aneurysm domain to a 0D or 1D lumped parameter network representing the global cardiovascular system (Moghadam et al., 2013). While such 0D-3D or 1D-3D coupling could potentially better account for the effect of patient-specific geometries on the inlet flow profile, it may be difficult to identify patient-specific values for the parameter values of the network, and the estimation of said parameters warrants careful thought.

Further, we categorized aneurysms as stable or growing based upon imaging studies separated in time by at least 1 year, but it is possible that aneurysms in this study have been miscategorized. For instance, aneurysms that seem to be stable over a 1-year period may in fact be growing aneurysms with a growth rate for which a 1-year time interval was not enough to detect any change. Including growing aneurysms in our control group would inhibit our ability to detect differences between groups.

Finally, we were limited by the low cohort size in this matched-pair study. The inclusion of additional patients could increase the statistical power of our findings and may be particularly impactful in investigating metrics we observed at *p* = 0.10 significance. This points to future work where our modeling pipeline could be applied to a larger cohort of matched-pair cerebral aneurysms. In addition, future studies could investigate a wider variety of hemodynamic metrics. While our study focused on the relationship between WSS and aneurysm growth due to the heavy implication of WSS as a driving factor of aneurysm progression, several studies have found significant or near-significant correlation between vorticity and shear concentration and aneurysm growth and rupture (Sforza et al., 2016; Brinjikji et al., 2017). Applying our robust modeling techniques to a study where these additional metrics are investigated would increase confidence in the analyzed hemodynamic values.

## Data Availability

The raw data supporting the conclusion of this article will be made available by the authors, without undue reservation.
