# Supplementary material for: A matched-pair case control study identifying hemodynamic predictors of cerebral aneurysm growth using computational fluid dynamics
Source: Front Physiol. 2023 Dec 15;14:1300754. doi: 10.3389/fphys.2023.1300754 (PMC10757566; doi:10.3389/fphys.2023.1300754)
Supplement: Supplementary file 1 [file DataSheet1.pdf]

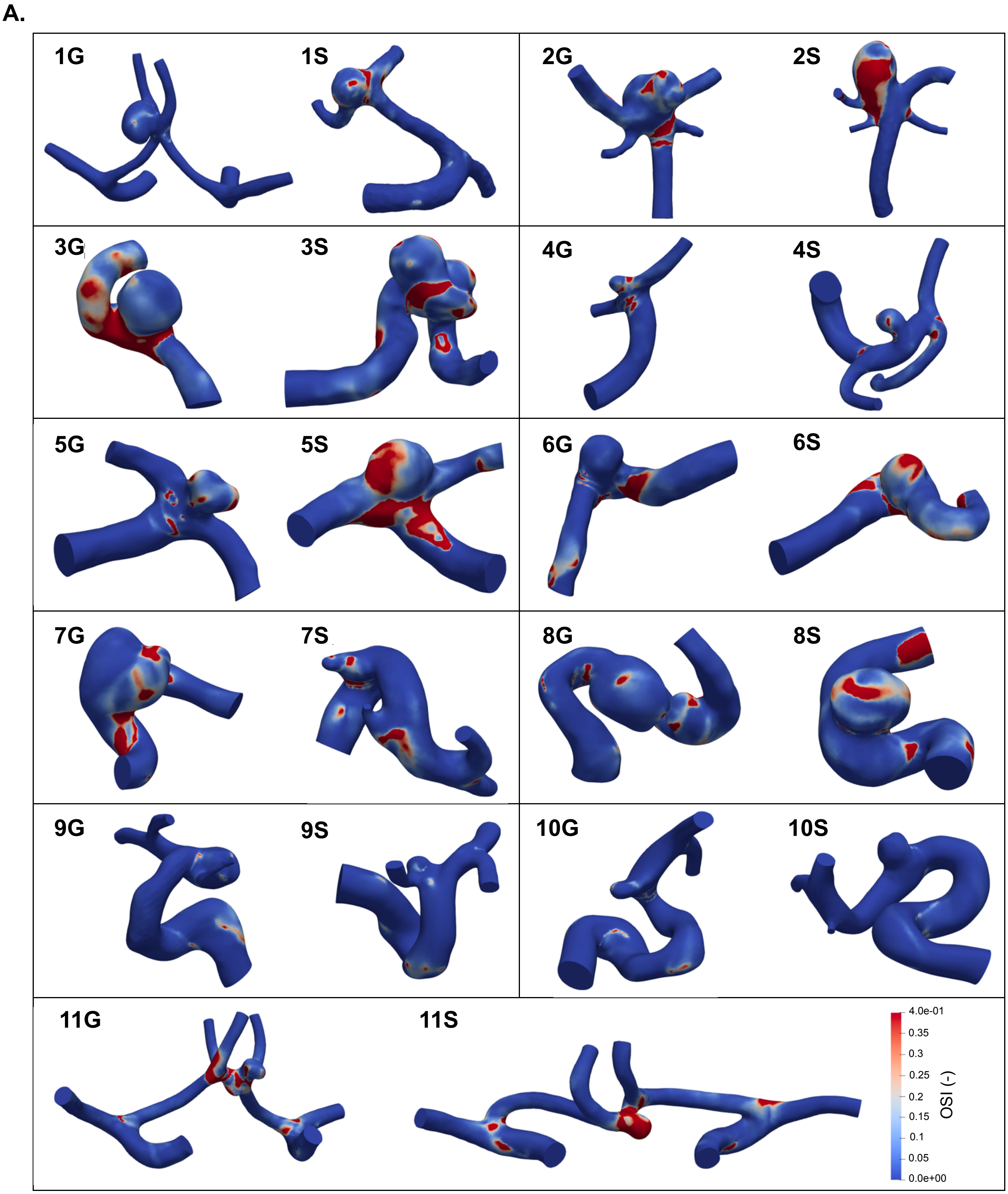
A.
1G
1S
2G
2S
3G
3S
4G
4S
5G
5S
6G
6S
7G
7S
8G
8S
9G
9S
10G
10S
11G
11S
4.0e-01
0.35
0.3
0.25
0.2
0.15
0.1
0.05
0.0e+00
OSI (-)

B.

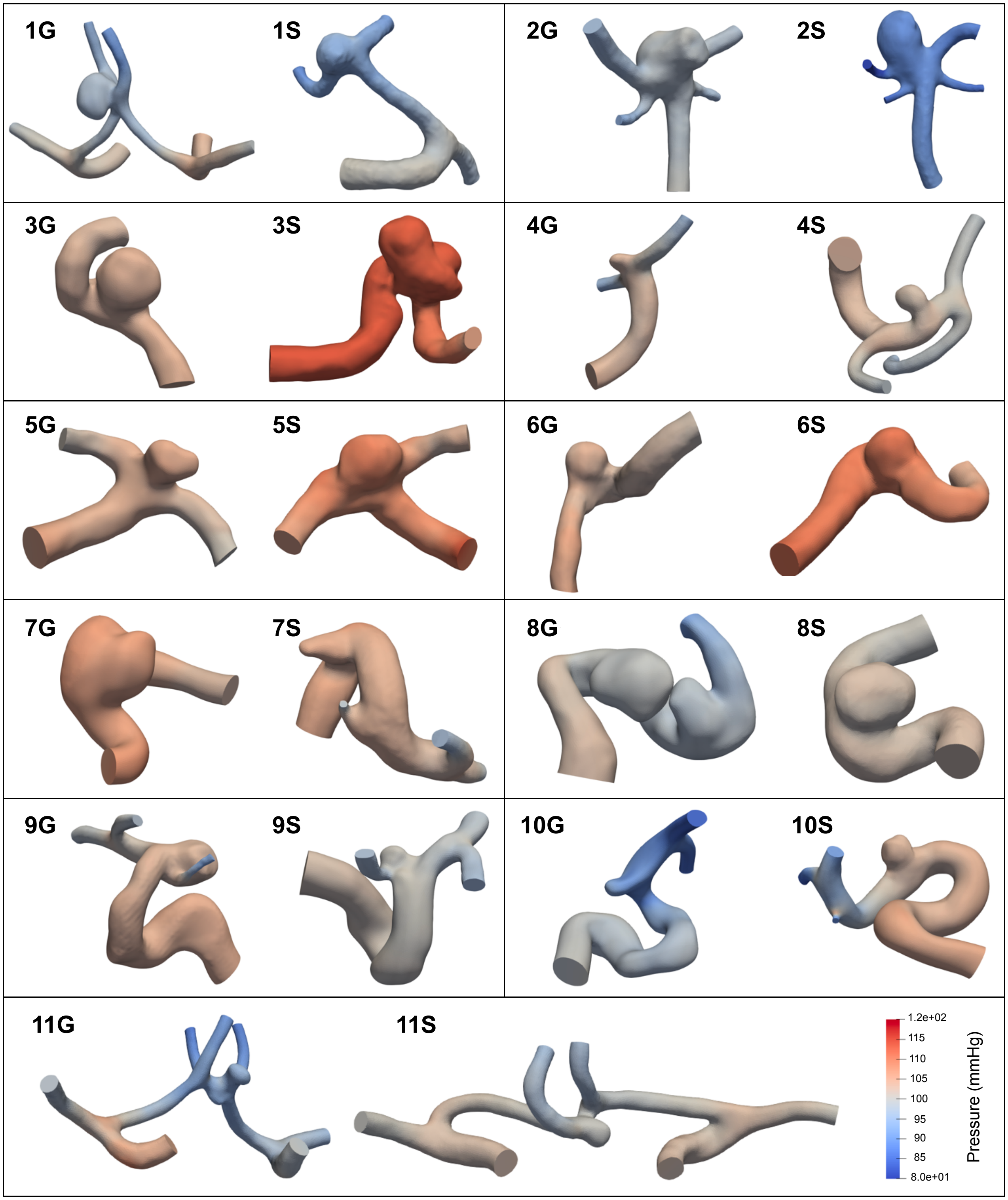

Supplementary Figure 1: (A) OSI distributions and (B) pressure distributions of each of the 11 pairs of aneurysms in our cohort. All pressures are in mmHg.

Supplementary Table 1: RCR values of each of our simulated models, used for our final, converged simulations.

Model	*Vessel*	R_p	*C*	R_d	*Model*	*Vessel*	R_p	*C*	R_d
1G	ACA 1	5.58E3	3.85E-5	5.02E4	*1S*	ACA 1	8.17E3	2.27E-6	7.35E4
	ACA 2	9.04E3	1.60E-6	8.13E4		ACA 2	1.46E4	1.27E-6	1.31E5
	MCA 1	5.40E3	2.69E-6	4.86E4		MCA	6.26E3	2.96E-6	5.63E4
	MCA 2	4.66E3	3.11E-6	4.20E4		-	-	-	-
2G	PCA 1	1.17E3	4.59E-6	1.05E4	*2S*	PCA 1	7.27E3	5.16E-5	6.55E4
	PCA 2	1.06E3	5.06E-6	9.56E3		PCA 2	1.51E4	2.49E-5	1.36E5
	SCA 1	3.34E3	1.61E-6	3.01E4		SCA 1	2.64E4	1.42E-5	3.54E5
	SCA 2	4.32E3	1.25E-6	3.89E4		SCA 2	3.93E4	9.543E-6	3.54E5
3G	ICA	3.38E3	6.60E-6	3.04E4	*3S*	ICA	3.48E3	6.0E-6	3.13E4
4G	MCA	4.68E3	1.55E-6	4.21E4	*4S*	ACA	1.12E4	9.36E-6	1.01E5
	PCOMM	7.62E3	6.57E-6	6.86E4		MCA	6.01E3	1.248E-6	5.41E4
	-	-	-	-		PCOMM	1.28E4	5.84E-7	1.16E5
5G	ACA	8.86E3	3.0E-6	7.97E4	*5S*	ACA	6.14E3	3.5E-6	5.53E4
	MCA	5.50E3	3.0E-6	4.95E4		MCA	7.68E3	3.5E-6	6.91E4
6G	ICA	3.30E3	6.0E-6	2.97E4	*6S*	ICA	3.42E3	6.5E-6	3.08E4
7G	ICA	3.34E3	7.43E-6	3.01E4	*7S*	ACA	7.92E3	3.01E-6	7.13E4
	-	-	-	-		MCA	6.01E3	3.97E-6	5.41E4
	-	-	-	-		OA	4.53E4	5.26E-7	4.08E5
8G	ICA	2.98E3	7.43E-6	3.01E4	*8S*	ICA	3.27E3	6.02E-6	2.94E4
9G	ACA	1.33E4	1.79E-6	1.19E5	*9S*	ACA	1.33E4	1.55E-6	1.20E5
	MCA	4.98E3	4.79E-6	4.48E4		MCA	5.57E3	3.71E-6	5.01E4
	PCOMM	2.60E4	9.15E-7	2.34E5		PCOMM	1.66E4	1.24E-6	1.50E5
10G	ACA	9.93E3	4.03E-6	8.94E4	*10S*	ACA	4.88E3	7.68E-6	4.39E4
	MCA	3.82E3	1.05E-5	3.44E4		MCA	9.31E3	4.03E-6	8.38E4
	-	-	-	-		OA	2.93E4	1.28E-6	2.64E5
11G	ACA 1	8.80E3	6.18E-5	7.92E4	*11S*	ACA 1	6.73E3	3.14E-5	6.06E4
	ACA 2	1.34E4	1.06E-6	1.21E5		ACA 2	6.19E3	1.70E-6	5.57E4
	ACA 3	1.15E4	1.24E-6	1.04E5		MCA 1	5.07E3	2.07E-6	4.56E4
	MCA 1	5.37E3	2.65E-6	4.83E4		MCA 2	6.27E3	1.67E-6	5.65E4
	MCA 2	4.16E3	3.43E-6	3.74E4		-	-	-	-

Supplementary Table 2: Mean and standard deviations of hemodynamic variables of interest for stable and growing aneurysms, and corresponding p values from the paired Wilcoxon rank sum test.

	Stable		*Growing*		*p Value*
	Mean	*SD*	*Mean*	*SD*	
Mean TAWSS (dyne/cm²)	55.60	29.82	72.74	68.78	0.83
Max TAWSS (dyne/cm²)	140.08	61.20	186.50	134.19	0.41
Min TAWSS (dyne/cm²)	11.14	7.74	11.38	10.83	0.97
$\frac{Mean\ dome\ TAWSS}{Mean\ parent\ TAWSS}$	0.73	0.38	0.54	0.23	0.21
$\frac{Mean\ dome\ OSI}{Mean\ parent\ OSI}$	9.93	13.92	0.01	0.009	0.70
Mean OSI	0.03	0.05	0.01	0.01	0.41
Max OSI	0.27	0.12	0.25	0.08	0.83
Min OSI	0.002	0.003	0.0005	0.0003	0.41
LSA (%)	46.72	33.23	36.92	38.12	0.46
$MTLOSIA_{10\%}$ *(%)*	7.25	17.05	8.65	16.11	1.00
$MTLOSIA_{30\%}$ *(%)*	15.72	24.99	24.71	33.79	0.65
$MTLOSIA_{50\%}$ *(%)*	22.85	28.43	31.96	36.55	0.65
$MTLOSIA_{70\%}$ *(%)*	27.01	30.30	37.42	37.58	0.64
$MTLOSIA_{90\%}$ *(%)*	30.62	31.85	41.69	37.52	0.52
$MTLSA_{10\%}$ *(%)*	7.25	21.12	7.02	12.47	0.48
$MTLSA_{30\%}$ *(%)*	19.97	29.95	28.98	28.95	0.28
$MTLSA_{50\%}$ *(%)*	37.60	33.07	52.00	28.11	0.10
$MTLSA_{70\%}$ *(%)*	55.74	30.59	73.30	23.21	$0.08^{\dagger}$
$MTLSA_{90\%}$ *(%)*	69.81	26.38	87.22	17.14	0.11

Abbreviations: TAWSS, time averaged wall shear stress; OSI, oscillatory shear index; LSA, low shear area; MTLOSIA, mean-thresholded low OSI area; MTLSA, mean-thresholded low shear area

Supplementary Table 3: Results of the Shapiro-Wilk test for normality of hemodynamic variables of interest for stable and growing aneurysms, and corresponding p values, where $\alpha = 0.05$ and the null hypothesis is that the data is normally distributed.

	Stable		*Growing*	
	Normal	*p Value*	*Normal*	*p Value*
Mean TAWSS (dyne/cm^2)	Yes	0.50	No	9.33E-4
Max TAWSS (dyne/cm^2)	Yes	0.36	Yes	0.16
Min TAWSS (dyne/cm^2)	Yes	0.39	No	3.17E-3
Mean OSI	No	2.73E-6	Yes	0.93
Max OSI	Yes	0.41	Yes	0.41
Min OSI	No	1.59E-4	Yes	0.22
LSA (%)	Yes	0.55	No	0.027
$MTLSA_{50\%}$ (%)	No	0.027	Yes	0.95
$MTLSA_{70\%}$ (%)	Yes	0.46	Yes	0.16
$MTLSA_{90\%}$ (%)	Yes	0.17	No	4.09E-3
